# Native SAD is maturing

**DOI:** 10.1107/S2052252515008337

**Published:** 2015-05-14

**Authors:** John P. Rose, Bi-Cheng Wang, Manfred S. Weiss

**Affiliations:** a Department of Biochemistry and Molecular Biology, University of Georgia, Athens, Georgia, USA; b Helmholtz-Zentrum Berlin für Materialien und Energie, Berlin, Germany

**Keywords:** native SAD, sulfur SAD, accurate data collection, data multiplicity, radiation damage, new instruments, new data-scaling techniques

## Abstract

Native SAD phasing uses the anomalous scattering signal of light atoms in the crystalline, native samples of macromolecules collected from single-wavelength X-ray diffraction experiments. These atoms include sodium, magnesium, phosphorus, sulfur, chlorine, potassium and calcium. Native SAD phasing is challenging and is critically dependent on the collection of accurate data. Over the past five years, advances in diffraction hardware, crystallographic software, data-collection methods and strategies, and the use of data statistics have been witnessed which allow ‘highly accurate data’ to be routinely collected. Today, native SAD sits on the verge of becoming a ‘first-choice’ method for both *de novo* and molecular-replacement structure determination. This article will focus on advances that have caught the attention of the community over the past five years. It will also highlight both *de novo* native SAD structures and recent structures that were key to methods development.

## Abstract 2

Advancements in source stability, X-ray optics, detectors, goniometry, data-collection strategies and data reduction and phasing software made over the past decade have placed native SAD at the verge of becoming a ‘first-choice’ method for both *de novo* and molecular-replacement structure determination.

## 1. Introduction   

Native SAD phasing uses the anomalous scattering signal of atoms in the crystalline, native samples of macromolecules collected from single-wavelength X-ray diffraction experiments. These atoms include sulfur and some other light atoms found in native proteins and DNA, RNA or buffer. Compared with metals, the anomalous scattering signal from these light atoms is relatively small. In the tunable range of most synchrotrons (17–6 keV) the anomalous scattering signal of sulfur, as defined by Δ*f*′′, ranges from 0.13 to 0.95 e^−^. The phosphorus signal in this range is smaller, ranging from 0.10 to 0.75 e^−^. In comparison, the iron signal ranges from 0.89 to 3.95 e^−^ at 7.15 keV (the iron absorption edge). The anomalous scattering signal for zinc, another metal commonly found in proteins, ranges from 1.50 to 3.79 e^−^ (or more owing to white-line effects) at 9.66 keV (the zinc absorption edge). Thus, with the exception of metalloproteins, native SAD phasing is critically dependent on accurately recording the weak anomalous scattering signal from light atoms such as sulfur, phosphorus, chlorine, potassium, calcium and magnesium present in the crystallized sample. This requires special attention to all aspects of the experiment from sample preparation to phasing focused on mitigating or eliminating all sources of noise in the process in order to increase the anomalous signal-to-noise ratio in the data. This review will focus on *de novo* and other important native SAD structures (148 in total; see Supplementary Table S1) reported in the Protein Data Bank (PDB; Berman *et al.*, 2000 ▸) that do not contain atoms heavier than calcium (atomic number 20), hereafter termed native SAD structures, and recent advances in the method.

The challenge of resolving the phase ambiguity associated with single-wavelength data and accurately recording the anomalous scattering signal of these light atoms is reflected by the fact that while the first native SAD structure, that of crambin, was reported in 1981 (Hendrickson & Teeter, 1981 ▸), it took almost 20 years until the structure of hen egg-white lysozyme was redetermined by sulfur SAD (S-SAD; Dauter *et al.*, 1999 ▸) and the second *de novo* S-SAD structure, that of obelin (Liu *et al.*, 2000 ▸), was reported. Both structures were determined using a new phasing approach developed by B.-C. Wang (Wang, 1985 ▸).

Wang’s process, commonly known as solvent flattening, involved first identifying the molecular envelope (or solvent boundary) of the protein followed by solvent flattening *via* carrying out iterative rounds of a reciprocal-space (phase) and real-space (density) noise-filtering process to produce the final set of experimental SAD phases for structural analysis. The method was a key advance since it addresses two critical bottlenecks in the S-SAD phasing method introduced for the determination of crambin: (i) the phase-ambiguity problem in using SAD data and (ii) the requirement that the protein must have a large sulfur content as in crambin (six S atoms in 46 residues or 13% Cys + Met content), which is atypical of most proteins. In his 1985 paper, Wang also showed the potential of the approach for S-SAD phasing through a proof-of-concept computer simulation using error-free anomalous data showing that the Bence–Jones protein Rhe (two S atoms in 113 residues) could be successfully phased using only the anomalous scattering signal from a single disulfide bond.

The removal of these bottlenecks was key to the structure determination of the second *de novo* S-SAD structure 20 years later. The method has been shown to be generally applicable to all SAD data (Au-SAD, Wang, 1985 ▸; I-SAD, Chen *et al.*, 1991 ▸), and for S-SAD it marked the beginning of structure determination of proteins having a more typical (∼3%) Cys + Met content.

Although both the theoretical and practical aspects of a successful S-SAD phasing were clearly demonstrated by the structure determination of obelin, which had a more typical sulfur content of eight S atoms in 189 residues, it took another 15 years for native SAD to become a fast and practical method for structure determination as reported in recent publications.

Over the past 15 years, we have witnessed some tremendous advances in diffraction hardware, crystallographic software, data-collection methods and strategies and the use of data statistics, which allow ‘highly accurate data’ to be routinely collected. This article will focus on advances that have caught the attention of the community. It will highlight both *de novo* native SAD structures and recent structures that were key to recent methods development. A fully comprehensive review is in the planning stage and hopefully will be a future follow-up article with additional information contributed from the community.

## 2. Current state of the art   

**Figure 1:**
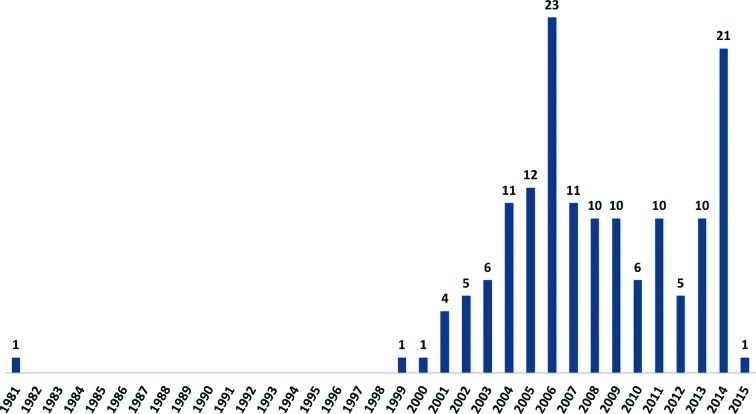
A plot of the number of native SAD structures deposited per year in the Protein Data Bank. The number shown include both *de novo* structures and previously solved structures that have been redetermined as part of software and methods development. Excluded from the list are standard proteins such as insulin, lysozyme, thaumatin, trypsin and glucose isomerase. The peak in native SAD structure determinations during the period from 2004 to 2009 reflects, among other things, the introduction of the Rigaku chromium-rotating anode, which was used by the SECSG (Adams *et al.*, 2003 ▸) and SGC (Yakunin *et al.*, 2004 ▸) structural genomics centers, and the work carried out by Cheng Yang at Rigaku and Nobuhisa Watanabe at Hokkaido University, Japan. The large spike in PDB entries in 2006 reflects ten structures reported in a methods-development paper (Mueller-Dieckmann *et al.*, 2007 ▸), which first noted that chloride, sulfate, phosphate or metal ions present in the crystal can contribute to the anomalous signal in the data. The large increase in PDB depositions for 2014 reflects the proteins used for the development of multi-data-set averaging (from single or multiple crystals) methods, with 11 structures representing the SLS studies reported in 2014 (Weinert *et al.*, 2015 ▸).

Today, there are close to 150 *de novo* native SAD structures in the Protein Data Bank (Fig. 1 ▸), which recently announced its 108 000th structure. However, advances in technology and methodology during the past five years in the areas of X-ray sources, detectors, sample preparation, data-collection strategies, data reduction, phasing and structure solution, as discussed below, show great promise in making native SAD phasing a routine approach for macromolecular structure determination.

### 2.1. Sources   

**Figure 2:**
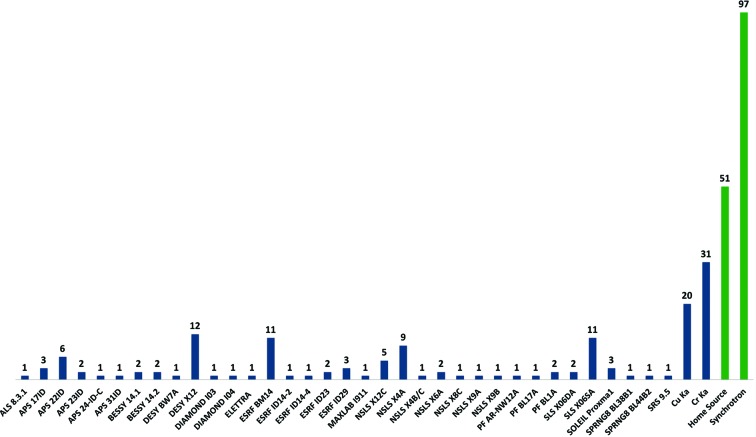
A plot of the number of native SAD structures deposited in the Protein Data Bank by X-ray source. Home-source structures include 20 from copper sources (λ = 1.5418 Å) and 31 from chromium sources (λ = 2.2909 Å). The last five years have seen a dramatic rise in synchrotron structures, while home-source structures remain flat.

**Figure 3:**
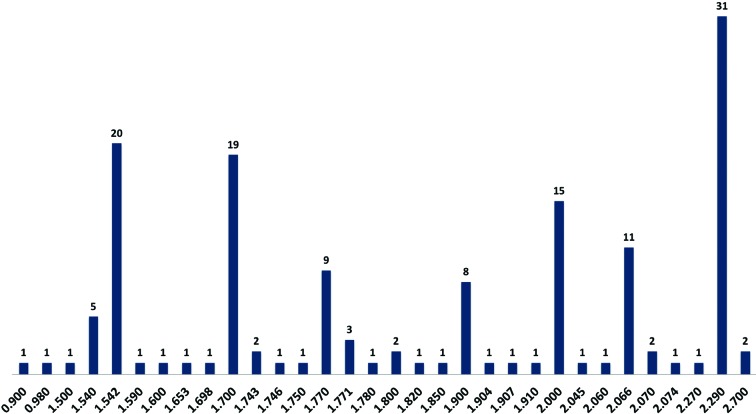
A plot of the number of native SAD structures deposited in the Protein Data Bank as a function of the wavelength used for data collection. The large peaks at 1.542 and 2.290 Å represent copper and chromium home sources, respectively. The two structures for which data were recorded with 2.7 Å X-rays come from BL-1A at the Photon Factory.

One advantage of native SAD phasing is that it is not dependent on a tunable X-ray source or access to an X-ray absorption edge. For example, the crambin data were collected using a sealed-tube copper X-ray source [λ = 1.5418 Å; Δ*f*′′(S) = 0.56 e^−^] and a four-circle diffractometer. Today, about 35% (52) of the native SAD structures reported (*i.e.* structures that have a Protein Data Bank ID) have been determined from data collected in-house, with 20 structures being determined using copper X-rays and 31 structures determined using chromium X-rays [λ = 2.2909 Å; Δ*f*′′(S) = 1.15e^−^] (Rose *et al.*, 2004 ▸), including the 84 kDa α-glucosidase SusB (PDB entry 2d73) containing two molecules per asymmetric unit (Kitamura *et al.*, 2008 ▸). The remaining 96 structures were determined using synchrotron data. It should be noted that most of the beamlines presented in Fig. 2 ▸ were designed and optimized to support MAD data collection at the selenium absorption edge (λ = 0.9795 Å) and the collection of high-resolution data, which represent the majority of the experiments carried out on these beamlines. Native SAD phasing generally requires data collection using X-ray wavelengths away from the selenium absorption edge, where beam stability and X-ray absorption can become problematic. An analysis of native SAD structures determined from synchrotron data (Fig. 3 ▸) shows that a majority of these structures result from data recorded using 1.7–1.8 Å X-rays, reflecting a compromise between the increase in the anomalous scattering signal for light atoms (higher signal) and the increase in X-ray absorption and beam instability (higher noise) as the wavelength increases.

**Figure 4:**
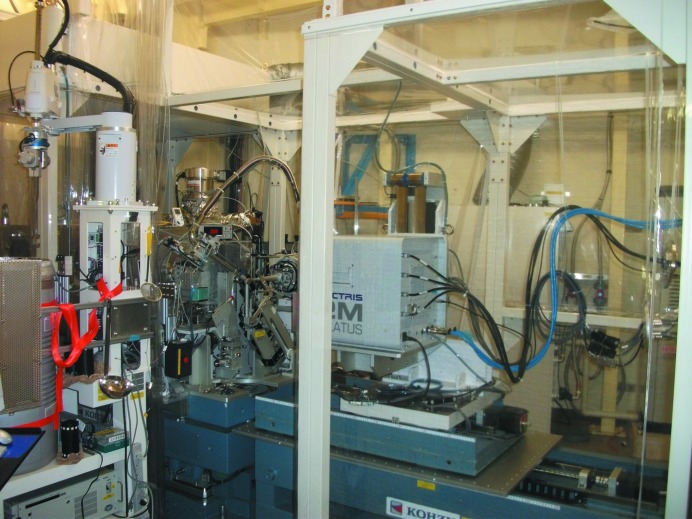
A photograph of the long-wavelength beamline BL-1A at the Photon Factory (courtesy of Naohiro Matsugaki). The beamline has been optimized for data collection using X-rays in the range from 2.7 to 3.5 Å, with the entire experimental station enclosed in a helium box to reduce air absorption at longer wavelengths.

To address the X-ray absorption and beam-stability issues encountered when longer wavelengths are used, researchers at the Photon Factory in Japan and the Diamond Light Source in the United Kingdom have built the first dedicated beamlines for native SAD data collection. Using an in-vacuum short-gap undulator and optimized optics to provide stable X-ray micro-beams and enclosing critical end-station components (beam port, goniometer, detector and cryostream) in a helium-filled chamber to reduce absorption, Photon Factory beamline BL-1A (Fig. 4 ▸) has been designed to provide stable long-wavelength X-rays in the range 2.7–3.3 Å. During the commissioning of the beamline, the native SAD structure of the ectodomain of death receptor 6 (34.1 kDa; 21 S atoms) was determined using 2.7 Å X-rays [Δ*f*′′(S) = 1.52 e^−^; Ru *et al.*, 2012 ▸]. More recently, BL-1A data collected using 2.7 Å X-rays enabled the native SAD structure solution of a lipocalin-like protein (18.7 kDa; five S atoms) using crystals harvested from the cockroach midgut (PDB entry 4nyr; N. P. Coussens, F.-X. Gallat, S. Rama­swamy, K. Yagi, S. S. Tobe, B. Stay & L M. G. Chavas, unpublished work). The structure is significant in that it represents the first case of a native SAD structure being determined from triclinic crystals.

**Figure 5:**
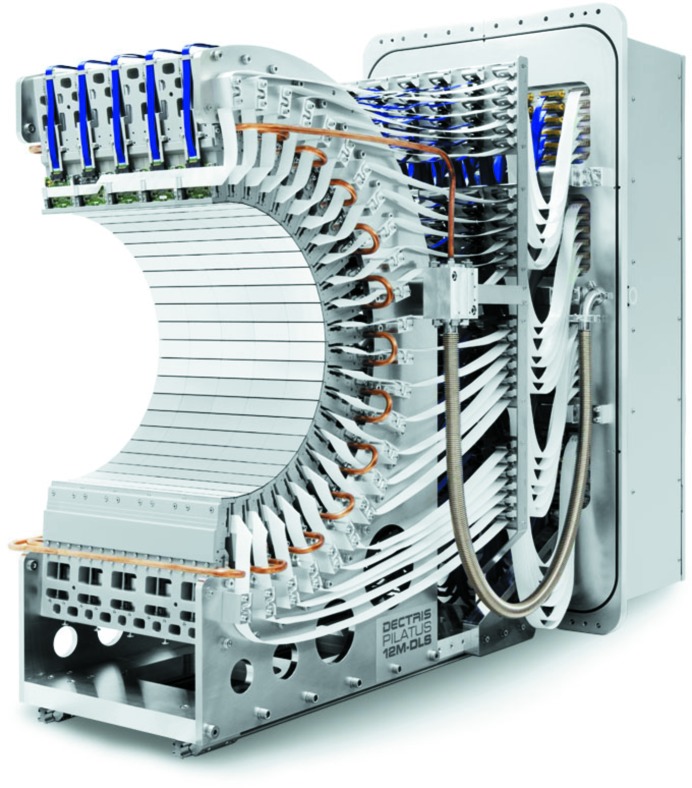
A photograph of the PILATUS 12M detector (courtesy of DECTRIS) installed on beamline I23 at the Diamond Light Source. The custom curved detector will be used to collect native SAD data up to the sulfur edge (λ = 5.01 Å), where the sulfur anomalous signal Δ*f*′′ is 4.1 e^−^. The detector has been designed to operate in a vacuum since the entire I23 endstation sits in a vacuum vessel to reduce air absorption. The curved detector allows access to diffraction data up to 2θ = ±100°.

In the United Kingdom, researchers are commissioning beamline I23 at the Diamond Light Source for long-wavelength crystallography. The beamline has been specifically designed for native SAD experiments and will provide stable X-rays in the range from 1.5 to 4 Å [Δ*f*′′(S) = 3.06 e^−^]. To reduce X-ray absorption and scattering effects, the entire experiment will be carried out *in vacuo* using the DECTRIS PILATUS 12M, a large semi-cylindrical hybrid photon-counting detector (Marchal & Wagner, 2011 ▸) designed to reduce parallax at these wavelengths (Fig. 5 ▸). Frozen crystals will be introduced into the vacuum chamber and mounted using a custom magnetic joint-based sample holder adapted from similar devices used in cryo­electron microscopy (Mykhaylyk & Wagner, 2013 ▸). X-ray tomography will be used to determine the dimensions of the crystal for empirical absorption corrections. The first data sets from the beamline are expected in early 2015.

### 2.2. Detectors   

The recent introduction of fast detectors such as the DECTRIS PILATUS/EIGER hybrid photon-counting detectors (http://www.dectris.com; Broennimann *et al.*, 2006 ▸) and the CCD-based Rayonix HS series of detectors (http://www.rayonix.com) at beamlines around the world has significantly impacted the ease with which native SAD data collection can be carried out. The fast (10 to 1000 Hz or greater) detectors with readout times of 1 ms or better enable shutter­less data collection, reducing the noise associated with shutter synchronization error. These detectors also allow efficient fine-sliced data collection, reducing background fog on the image, which increases the anomalous signal to noise in the data.

The hybrid photon-counting detectors introduced in 2006 offer the advantage of high (20 bit) dynamic range, zero read noise and a ‘top-hat’ point-spread function with pixel sizes ranging from 172 µm (Dectris PILATUS) to 75 µm (Dectris EIGER), while the recently introduced fast CCD-based detectors are integrating detectors (16 bit) and offer selectable frame rates ranging from 10 Hz (2 × 2 binning, 78 µm pixels) to 55 Hz (5 × 5 binning, 195 µm pixels).

A dual-mode pixel-array detector is currently being developed by ADSC (http://www.adsc-xray.com), which will support both photon-counting and photon-accumulation (charge-ramp counting) modes at frame rates (22 bit, 150 µm pixels) of up to 50 Hz (200 Hz with the optional high-throughput computing server). In photon-counting mode the detector can support a maximum signal of four million 12 keV photons per pixel, while in charge-ramp accumulation mode the maximum signal per pixel is increased to 200 million 12 keV photons per pixel. Since the counting modes on the detector can be selected on a pixel-by-pixel basis, the photon-counting mode can be used to record the weaker high-resolution data, while charge-ramp counting mode, with its higher saturation level, can be used to record the more intense low-resolution data. The first ADSC instrument is currently being tested.

### 2.3. Sample preparation   

The native SAD experiment is critically dependent on eliminating all sources of noise in the process. This includes using crystals of the highest diffraction quality (*e.g.* diffraction resolution and mosaicity), selecting the proper size and material for the cryoloop and optimizing cryoprotection.

#### 2.3.1. Crystals   

Generally speaking, the better your crystal diffracts the better the data collected from it, increasing the success rate of the native SAD experiment. Thus, a little time spent in the laboratory optimizing crystals (and cryoprotectant cocktails) can often lessen the amount of time and data needed to solve the structure.

#### 2.3.2. Pins and loops   

Several pin–loop designs are commercially available for harvesting and mounting crystals for data collection at cryogenic temperatures, but care must be used when deciding which pin–loop design is best for the native SAD experiment. Recent studies (Alkire *et al.*, 2008 ▸, 2013 ▸) illustrate the effect of pin–loop design on data quality. The authors recommend that when choosing a pin–loop design the loop stem (the area between the pin head and loop) should be as short as possible and that the diameter of the loop should be chosen to fit the size of the crystal. Additionally, when nylon loops are used they recommend reinforcing the loop stem with epoxy or grease to reduce or eliminate vibration of the loop in the cold stream during data collection. This is especially important for native SAD experiments or when data are collected at high speeds (rates of >2 Hz).

The scatter from the loop and the solution that it contains is another source of noise in the experiment. Several methods have been developed to address this source of noise. In the loopless mounting method (Kitago *et al.*, 2010 ▸), a specially designed pin–loop assembly is used to harvest the crystal. Next, the solution surrounding the crystal is removed by aspiration *via* a channel running through the pin and the crystal is quickly flash-cooled in a cryogenic nitrogen-gas stream. The loop is then carefully removed using a small hook or forceps, leaving the crystal mounted directly on the pin ready for data collection. An alternate method uses a laser to vaporize the loop and then shape the crystal into a sphere to reduce absorption effects (Watanabe, 2006 ▸).

Wierman *et al.* (2013 ▸) have recently reported the use of graphene-wrapped crystals to maintain crystal humidity and reduce X-ray scatter. In this approach, a small sheet of multilayer (3–5 layers) graphene is floated on the bottom of a droplet of the mother liquor of the crystal suspended in a 1–2 mm loop. The crystal is then positioned in the drop on the hydrated side of the graphene sheet. A pin–loop assembly is then inserted into the droplet, centered above the crystal and dragged through the bottom of the droplet, wrapping the crystal (and loop) in graphene and trapping a small amount of mother liquor with the crystal. Preliminary data show that the multilayer graphene sheets are essentially transparent to X-rays and that scatter from the graphene-wrapped crystal is significantly lower when compared with traditional loop-mounted crystals. Another advantage of graphene wrapping is that it prevents dehydration of the crystal during data collection, allowing room-temperature data sets to be collected.

Another approach uses crystallization in ionically cross-linked polysaccharide gel beads to reduce mechanical damage to crystals during mounting and osmotic shock during cryoprotection (Sugahara, 2014 ▸). In this method, crystals are grown inside an ionically cross-linked polysaccharide gel bead using the microbatch-under-oil technique. The aqueous protein–polysaccharide solution containing either 2%(*w*/*v*) alginate or 1.5%(*w*/*v*) k-carrageenan is introduced into the paraffin oil layer covering a well of Nunc HLA crystal plate containing the precipitant cocktail plus either the calcium (alginate) or potassium/sodium (k-carrageenan) ions needed to initiate the cross-linking reaction. When the protein–polysaccharide drop enters the aqueous precipitant cocktail the cross-linking reaction begins immediately, forming a gel bead with a diameter of 0.5–0.9 mm.

The setup is then incubated at 293 K until crystals are observed. The crystal-containing gel beads are harvested *via* a vacuum tweezer (Virtual Industries), mounted on a gonio­meter head and flashed-cooled in a cryogenic nitrogen-gas cold stream. The porous nature of the gel bead allows cryoprotection, ligand soaking and heavy-atom derivatization of the crystal as required. Initial tests using gel beads containing lysozyme crystals showed that the native SAD structure could be autosolved using data collected on a copper rotating-anode home source.

#### 2.3.3. Cryoprotection   

Today, most data are collected at cryogenic temperatures to reduce radiation damage using the loop-mounting technique (Teng, 1990 ▸), which reduces stress on the crystal during mounting. The cryocooling process can increase the crystal mosaicity, and cryoprotectants have been developed to limit this phenomenon. Non-optimal cryoprotection introduces noise in the native SAD experiment in the form of higher image backgrounds, reflection crowding (owing to high mosaicity) and the presence of ice rings in the pattern. Thus, cryoprotectant optimization is important for the native SAD experiment, and several excellent reviews have been written on this subject (see, for example, Garman, 2013 ▸). An alternate approach for crystal cooling using high-pressure (200 MPa) helium gas has been shown to provide excellent diffraction from noncryoprotected crystals (Kim *et al.*, 2005 ▸). The approach builds on myoglobin cryoprotection studies (Thomanek *et al.*, 1973 ▸) and is based on the phenomenon that water under high pressure freezes as ice III, which contracts as it freezes, compared with ice I, which expands. This technique has been used for the native SAD phasing of thaumatin crystals contained within a small capillary (Kim *et al.*, 2007 ▸). The high-pressure cooling process is similar to traditional loop cooling except that no cryoprotectant is used and the loop-mounted crystal is coated in oil to prevent dehydration. The crystal is first incubated under high-pressure helium at room temperature for about 25 min and then dropped into the lower part of the pressure cylinder which has been cooled to cryogenic temperature. After waiting 10 min for the temperature of the crystal to reach 77 K, the pressure is released and the pin–loop assembly is transferred under liquid nitrogen to a cryovial and stored at cryogenic temperature for data collection. The technique has been further refined (Englisch *et al.*, 2011 ▸) and two commercial units are now available: the HPC-201 from Advanced Design Consulting USA (http://www.adc9001.com) and the HPM-010 system from BAL-TEC AG (http://www.bal-tec.com).

### 2.4. Data collection   

#### 2.4.1. Wavelength   

For the elements available for native SAD phasing, the anomalous scattering signal increases with increasing wavelength. However, X-ray absorption and beam-stability issues also increase. An optimal X-ray wavelength of 2.1 Å has been proposed for S-SAD phasing (Mueller-Dieckmann *et al.*, 2005 ▸), but until recently few structures had been reported using synchrotron data collected at wavelengths above 2 Å (see Fig. 3 ▸), with the majority of synchrotron structures determined using X-ray wavelengths close to the iron absorption edge (λ = 1.74 Å) in the range 1.7–1.8 Å. This observation would tend to support problems with beam stability at longer wavelengths, as discussed above, and not X-ray absorption, since 31 native SAD structures have been determined with home-source chromium X-rays (λ = 2.29 Å) and a helium beam path. Beam instability can be caused by several factors. Thermal deformations of optical components as the energy is changed can lead to beam drift as the system equilibrates. Mechanical vibrations of optical components can also be a factor, especially for microbeam/microcrystal experiments. Finally, positional instability of the electron-beam orbit within the synchrotron can also be a problem (Lesourd *et al.*, 2002 ▸). If thermal deformations are the problem, experimenters simply have to wait until the beam stabilizes after an energy change, which can be minutes or hours depending on the beamline design.

#### 2.4.2. Goniometry   

The native SAD experiment is also dependent on keeping the crystal centered in the X-ray beam during data collection. This task becomes more challenging as the crystal size and/or the beam size become smaller. Thus, careful crystal centering is essential to the success of the native SAD experiment. Modern goniometers such as the Bruker/ARINAX MD2/MD3 (http://www.bruker-est.com) installed on many beamlines around the world provide on-axis crystal viewing, which makes crystal centering much easier (Perrakis *et al.*, 1999 ▸). These goniometers also provide user-selectable pinholes to define beam size and other tools to help the user verify that the crystal is properly centered in the beam. Many beamlines also offer automated diffraction-based centering (rastering), where the crystal is translated in an *x*, *y*, *z* grid (step size dependent on beam size) and a diffraction image is recorded at each position using a highly attenuated X-ray beam (Hilgart *et al.*, 2011 ▸). These images are then used to determine the point (or points) of optimal diffraction, which can then be used to define the center of the crystal or hotspots for data collection.

Most beamline goniometers today offer the ability to translate the crystal during data collection, with the aim of reducing the possibility of radiation damage (Flot *et al.*, 2010 ▸). This is a very attractive feature since the native SAD experiment generally requires data sets with high reflection multiplicity, and radiation damage can present a problem. Two data-collection modes are generally provided: translational (or segmented) mode and helical mode. In segmented mode, the crystal is divided into domains (dependent on the crystal dimensions and the beam size). The length and direction of translation is determined by centering the crystal at the beginning and at the end of the desired translation vector. The data set is then collected along this vector beginning with domain 1 followed by domain 2 *etc.* until the data set is completed. The number of images collected in each segment depends upon the total number of images desired and on the number of domains available. Using the helical scan method, the length and direction of the translation vector is again defined by centering the crystal at the two end points, with the crystal being slowly translated along this vector during data collection. This mode offers the advantage (Zeldin, Gerstel *et al.*, 2013 ▸) of continually introducing fresh crystal into the beam during data collection.

In addition to the single ϕ-axis goniometers common to most beamlines, some facilities offer multi-axis goniometers such as the Bruker/ARINAX MD2/MD3 equipped with the MK3 mini-kappa goniometer or the PRIGo multi-axis goniometer recently developed at the Swiss Light Source (Waltersperger *et al.*, 2015 ▸). The key advantage of the multi-axis goniometer for the native SAD experiment is the ability to take advantage of crystal symmetry and/or habit to optimize the anomalous signal to noise during data collection (Brock­hauser *et al.*, 2013 ▸; Weinert *et al.*, 2015 ▸). This is achieved by aligning the twofold, fourfold or sixfold symmetry axis of the crystal along the spindle axis of the goniometer. This orientation allows Bijvoet mates to be measured at the same time from the same image. Data collection around a symmetry axis can also reduce the crystal rotation range needed to collect the complete data set, reducing the total X-ray dose that the crystal receives and the level of radiation damage during data collection. Finally, multi-axis goniometers can be used to reduce reflection density on images collected from crystals where one unit-cell axis is significantly longer than the other two. By orientating this axis parallel to the spindle during data collection, spot overlap can be minimized.

#### 2.4.3. Multiplicity   

Since the error associated with a measurement decreases with the square of the number of observations, it is common practice to collect data sets with high redundancy (or multiplicity) for the native SAD experiment (Cianci *et al.*, 2008 ▸; Weiss, 2001 ▸). Higher redundancy also improves the accuracy of the measurements, resulting in more detail in their associated electron-density maps and in the quality of the structure produced (Diederichs & Karplus, 1997 ▸). However, even at cryogenic temperatures the crystal has a finite lifetime in the beam before radiation damage occurs (Garman, 2013 ▸; Zeldin, Brockhauser *et al.*, 2013 ▸). Radiation damage is manifested by a loss of diffraction intensity during the course of the data collection. Structurally, this corresponds to the breakage of disulfide bonds, the loss of CO_2_ from aspartic and glutamic acid side chains and of the hydroxyl group from tyrosine, and the formation of free radicals. Thus, radiation damage can affect the outcome of S-SAD experiments in several ways. The theoretical dose needed to reduce the diffraction power of a protein by 50% has been calculated to be 2.2 × 10^7^ Gy (J kg^−1^; Henderson, 1990 ▸). This corresponds to a crystal lifetime ranging from 5.7 s to 11 h for an insertion-device beamline at the Advanced Photon Source using 12 keV X-rays (data taken from James Holton’s radiation-damage server; http://bl831.als.lbl.gov/~jamesh/ACA2007/damage_rates.pdf).

The theoretical half-life of the crystal in the X-ray beam can be calculated using *RADDOSE*-3*D* (Zeldin, Brockhauser *et al.*, 2013 ▸) based on the crystal composition and the beam parameters (size, shape, flux and energy). Experiments can then be designed to limit the effect of radiation damage.

#### 2.4.4. Strategies   

During the last three decades, SAD data-collection strategies have evolved from carefully designed protocols requiring data collection from aligned crystals using multi-axis goniometers to much simpler experiments on randomly orientated crystals with data collected around a fixed rotation axis. These early experiments generally employed the inverse-beam data-collection strategy (Hendrickson *et al.*, 1989 ▸), where the crystal is mounted along a symmetry axis and data are collected in small alternating data wedges (ϕ and ϕ + π) such that Bijvoet mates are collected on the same image and close together in time. This trend reflects advances in protein production (*e.g.* recombinant proteins and selenomethionine labeling), crystal mounting (loops), cryogenic data collection and hardware (synchrotron sources, X-ray optics, goniometry, detectors and computers) and improved software for data reduction and structure determination. Native SAD structure determination has followed a similar trend, with a majority of structures reported being determined using data collected with a single-axis goniometer and a randomly orientated crystal. However, as systems become more challenging researchers are going back to more sophisticated data-collection protocols, as outlined below.

A goal of native SAD data collection is to collect data sets of high multiplicity (to increase the anomalous signal-to-noise level in the data) without incurring significant radiation damage (a current bottleneck of the approach). Recently, two data-collection strategies have been reported that address this bottleneck: single-crystal low-dose multi-data set averaging (Liu *et al.*, 2011 ▸) and multi-crystal averaging (Liu *et al.*, 2012 ▸).

#### 2.4.5. The multi-crystal approach   

Multi-crystal averaging, as the name implies, involves averaging data sets collected from a number of different crystals in order to increase the reflection multiplicity of the final averaged data set. This strategy requires collecting data sets from a number of different crystals to minimize radiation damage. A cluster analysis of the individual processed data sets in terms of unit-cell deviation, diffraction dissimilarity and the calculated relative anomalous correlation coefficient (RACC) is then used to identify ‘outlier’ data sets and exclude them from the analysis (Liu *et al.*, 2013 ▸). The approach has been incorporated into both *BLEND* in *CCP*4 (Winn *et al.*, 2011 ▸; Foadi *et al.*, 2013 ▸) and *phenix.multi_crystal_average* (Adams *et al.*, 2010 ▸). Multi-crystal averaging has been applied to a wide range of proteins of varying sizes and complexity, including the integral membrane protein CysZ (498 residues; phased using 20 S atoms, four chloride ions and one sulfate ion; data collected from six crystals) from *Idiomarina loihiensis* (PDB entry 3tx3; New York Consortium on Membrane Protein Structure, unpublished work), the TorT–TorSS complex (1162 residues; phased using 28 S atoms and three sulfate ions; data collected from 13 crystals) from *Vibrio parahaemolyticus* (PDB entry 3o1i; Moore & Hendrickson, 2012 ▸) and the chaperone protein DnaK (1216 residues; phased using 32 S atoms, one sulfate ion and two ATP molecules; data collected from five crystals) from *Escherichia coli* (PDB entry 4jn4; Qi *et al.*, 2013 ▸).

Recently, this technique has been applied to native SAD structure determination of the *West Nile virus* NS1 protein (PDB entry 4tpl; Akey *et al.*, 2014 ▸). The crystals contained two NS1 monomers (754 residues, six disulfides, five methionine residues and one sulfate ion) per asymmetric unit and diffracted to 3.2 Å resolution. Data sets (two 90° ϕ, ϕ + π wedges) collected from 28 crystals were used in the analysis. After clustering, ten data sets were identified as outliers and excluded from the total set. Data for the remaining 18 crystals were then scaled and averaged, producing a 2.9 Å resolution data set containing 6 627 610 observations of 65 510 Bijvoet pairs with 100-fold multiplicity. The high multiplicity was critical to identifying the correct anomalous substructure. It also improved the map quality and extended the resolution limit for the weak data from ∼3.2 to 2.9 Å.

In another recent case, multi-crystal averaging was used to determine the native SAD structure of the N-terminal ectodomain domain of the *Hepatitis C virus* envelope protein E1 (PDB entry 4uoi; El Omari *et al.*, 2014 ▸). The crystals contained six ectodomain monomers (528 residues, ten disulfides) per asymmetric unit and diffracted to only 4.2 Å resolution. Data sets (two 45° ϕ, ϕ + π wedges) collected from 32 crystals were used in the cluster analysis with *BLEND*. Clustering showed that all 64 data wedges were consistent, and the data sets were merged to give a final data set containing ∼31 646 Bijvoet pairs with 121-fold multiplicity. Again the high multiplicity was critical to identifying the correct anomalous substructure since the useful anomalous scattering signal extended to only 6.5 Å resolution. Once the correct anomalous substructure had been identified, the structure was completed using *phenix.autosol*, which allowed the identification of several helices that were used to determine the noncrystallographic symmetry (NCS) operators. The structure was then completed using sixfold NCS averaging and phase extension using the 3.5 Å resolution native data set collected at 12.8 keV. This case is significant since it represents the lowest resolution native SAD structure determined to date.

#### 2.4.6. The single-crystal approach   

The single-crystal low-dose multi-data-set data-collection approach uses ‘dose-slicing’ to conserve the lifetime of the crystal. In this approach, multiple data sets are collected using one crystal with either an attenuated X-ray beam or reduced exposure time. The degree of attenuation or exposure-time reduction is inversely proportional to the number of data sets to be collected. For example, if the optimal exposure time for a given crystal is 1 s and one wished to collect three data sets, then the exposure time would be one-third of the optimal exposure time or one third of a second per image. The total X-ray dose accumulated for the three data sets is the same as the dose received for the data set collected using the optimal exposure time. The three data sets are then merged together to yield a final data set that will have an improved signal-to-noise ratio compared with that of the non-dose-sliced data set for a given X-ray dose. This is because the ‘dose-sliced’ multi-data set will give a smaller σ(*I*) value as indicated by the equation below, where the second term of the conventional sigma equation is divided by the number of data sets *N* (Liu *et al.*, 2011 ▸),
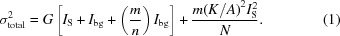



The improved *I*/σ values will further improve the Δ(*I*)/σ values of the strong reflections, which are important for native SAD phasing. This approach can easily be combined with either translation (or segmented) mode or helical mode data collection, which are already in practice for reducing radiation damage. Since the data have been collected from the same crystal, the data sets should be isomorphous if the exposure is kept below the Garman limit (Owen *et al.*, 2006 ▸). This is another advantage of the single-crystal approach.

Using the single-crystal dose-sliced multi-data set averaging strategy and the PRIGo multi-axis goniometer, a group from the Swiss Light Source has recently reported 11 native SAD structures determined on beamline X06DA using 6 keV X-rays (Weinert *et al.*, 2015 ▸). Generally, 3–5 dose-sliced data sets were collected at different crystal orientations and then merged to give the final data set. In this study, the initial anomalous substructure obtained from *SHELXD* (Sheldrick, 2010 ▸) was expanded using *Phaser* (Read & McCoy, 2011 ▸) and the sequence was autofitted to the resulting maps using *Buccaneer* (Cowtan, 2006 ▸) or *phenix.autobuild* (Adams *et al.*, 2010 ▸).

The structures reported include (i) mPGES1 (human microsomal prostaglandin E2 synthase 1; PDB entry 4wab), a 17 kDa integral membrane protein phased from nine S atoms and two chloride ions; (ii) DINB–DNA (*E. coli* DNA polymerase IV in complex with DNA; PDB entry 4r8u), a 98 kDa complex phased from 75 P atoms, 28 S atoms and two Ca^2+^ ions; (iii) the T2R–TTL multiprotein complex (a complex between αβ-tubulin, stathmin-4 and tubulin–tyrosine ligase; PDB entry 4wbn), a 266 kDa complex phased from 118 S atoms, 13 P atoms, three Ca^2+^ ions and two chloride ions. T2R–TTL is the largest native SAD structure determined to date.

### 2.5. Data processing   

The current generation of fast photon-counting and CCD detectors can collect data at an astonishing rate (10 Hz to 100 Hz or greater), and this coupled with ultrafine data slicing (*e.g.* 0.05° per frame) make data storage, data transfer and data reduction very demanding if not overwhelming in the home laboratory. A typical native SAD data set collected using these fast detectors could contain 25 000 images, depending on the rotation slice used. This large volume of data places demands on both disk space and processor speed. Transferring such a large volume of data over the internet is also challenging. Thus, many beamlines equipped with these fast detectors are providing computational resources at the facility for data reduction (either on-site or remotely), eliminating the need for extensive computing and data storage at the user’s home site. These beamlines in many cases have computer clusters that auto­process the data while they are being collected (Monaco *et al.*, 2013 ▸). It is important to note that although autoprocessing is fast and efficient, care must be used in the data-reduction process for native SAD data to ensure that the various processing parameters are optimally set.

Current data-reduction programs such as *XDS* (Kabsch, 2010 ▸), *HKL*-2000/*HKL*-3000 (Otwinowski & Minor, 1997 ▸; Minor *et al.*, 2006 ▸) and *MOSFLM* (Leslie & Powell, 2007 ▸) can all handle the ultrafine-sliced data sets produced by these new fast detectors. *XDS* offers the additional advantage of parallel data processing on systems having multiprocessors, thus speeding up the data integration.

### 2.6. Phasing and structure solution   

Phasing and structure solution can be broken down into three steps: (i) determining the anomalous substructure, (ii) determining the ‘hand’ of the data and (iii) inspecting the experimental electron-density map to determine whether the density makes sense (secondary structure present, side chains clearly defined *etc.*). Common software packages such as *CCP*4, the *SHELXC*/*D*/*E* suite (Sheldrick, 2010 ▸), *Auto-Rickshaw* (Panjikar *et al.*, 2005 ▸) and *PHENIX* can all be used carry out the native SAD phasing process.

Successful native SAD phasing is critically dependent on having the correct anomalous substructure. For example, in the case of the *West Nile virus* NS1 protein (Akey *et al.*, 2014 ▸), 100-fold Bijvoet multiplicity was required to obtain the correct anomalous substructure, but successful phasing only required 30–40-fold Bijvoet multiplicity. Both *SHELXD* and *phenix.hyss* can take advantage of multiprocessor or cluster-based systems to speed up the search for anomalous scatterers since, as was the case for the 20 kDa centromere M protein, over 50 000 *SHELXD* trials are sometimes needed to achieve the correct solution (Weinert *et al.*, 2015 ▸; Müller *et al.*, 2011 ▸).

Improved methods of solution ranking and phase generation have recently been reported. By introducing a SAD likelihood function to rank possible solutions from *phenix.hyss*, a study showed that in the case of CysZ multi-crystal data (Bunkóczi *et al.*, 2014 ▸) SAD likelihood ranking significantly improved the rate of success in finding the correct anomalous substructure in terms of both the strength of the anomalous signal and the number of crystals used to produce the merged data set. Another study introduces a direct phase-selection step prior to density modification (using *RESOLVE* or *DM*) that significantly improved the final experimental phases and the quality of the resulting electron-density maps (Chen *et al.*, 2014 ▸).

### 2.7. Building community   

**Figure 6:**
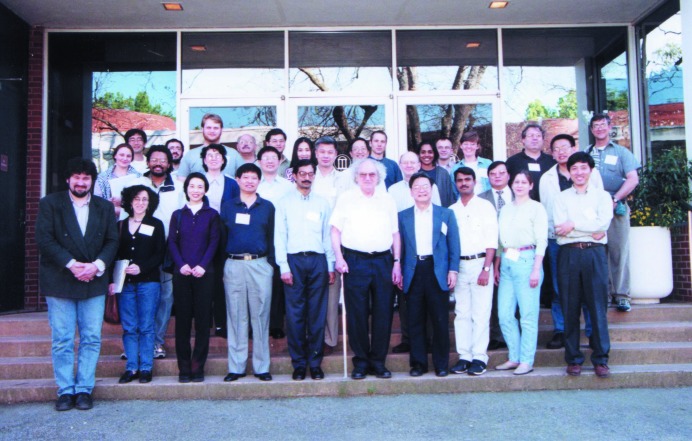
A group picture taken at the 2000 ISAS workshop held at the University of Georgia. The workshop attracted over 30 participants, including Herbert Hauptman, who wanted to learn how B.-C. Wang was solving S-SAD structures and especially how he was determining the handedness of the substructure.

Although the first native SAD structure was published in 1981 (Hendrickson & Teeter, 1981 ▸), almost 20 years passed before the S-SAD structure of the photoprotein obelin was published in 2000 (Liu *et al.*, 2000 ▸). The obelin structure was quickly followed by S-SAD structures of the Bence–Jones protein Len and PDO (Chen *et al.*, 2000 ▸) using the *ISAS* program (Wang, 1985 ▸). These achievements attracted considerable interest from the community, and a workshop on S-SAS phasing organized by B.-C. Wang was held at the University of Georgia in April 2000, which attracted over 30 participants (Fig. 6 ▸). Wang followed the first S-SAS workshop by workshops at the Annual Meeting of the American Crystallo­graphic Association in Los Angeles in July 2001 and at Tsinghua University in Beijing in June 2002. In 2003, the first Winter School on Soft X-rays in Macromolecular Crystallo­graphy was held in Bressanone/Brixen, Italy, with follow-up schools being held every three years since then. Over the past decade similar workshops and schools have been given at various locations worldwide, including MS 40: S-SAD and Other Applications of Soft X-rays in MX at the 2014 Congress of the International Union of Crystallography in Montreal and the fifth Winter School on Soft X-rays in Macromolecular Crystallography held at the University of Georgia in March 2015. These activities over the years have provided a forum where problems are discussed and new developments in hardware, software and methods are presented. In addition, they have brought together experts and interested parties to build a community that is dedicated to making native SAD the first choice for the *de novo* phasing of macromolecules.

## 3. Discussion   

Native SAD phasing is challenging and critically dependent on the collection of accurate data. To be considered as a routine or ‘first-choice’ phasing method, native SAD must meet the following criteria: (i) it must be no more difficult than Se-SAD, (ii) it must not require a special setup and (ii) a majority of the electron-density map should be autotraced (using *CCP*4, *PHENIX* or *SHELX*). In other words, native SAD must be easy to perform.

Dedicated long-wavelength beamlines are valuable to new approaches for native SAD. This is important since long-wavelength X-rays increase the anomalous scattering signal for the light atoms that are the focus of native SAD. The introduction of ultrafast detectors allows shutterless data collection and provides a means of efficient fine-sliced data collection, removing significant noise sources (shutter error and background fog, respectively) from the data. The use of multi-axis goniometers to take advantage of crystal habit to record Bijvoet pairs on the same image increases the accuracy of the Bijvoet difference, producing better data. The use of multi-data set averaging (either from a single crystal or from multiple crystals) has been shown to be a powerful way of increasing the Bijvoet multiplicity while limiting radiation damage, resulting in increasing the accuracy of the data and the strength of the anomalous signal to noise of the data set.

Taken together, the advances in source stability, X-ray optics, detectors, goniometry, data-collection strategies and data-reduction and phasing software made over the past decade have placed native SAD on the verge of becoming a ‘first choice’ method for both *de novo* and molecular-replacement structure determination. A community of dedicated scientists continually working on making native SAD easy to perform should make routine native SAD a reality.

## Supplementary Material

Supplementary Table S1. A listing of native SAD structures by year.. DOI: 10.1107/S2052252515008337/lz5007sup1.pdf
