# Supplementary material for: Native SAD is maturing
Source: IUCrJ. 2015 May 14;2(Pt 4):431–40. doi: 10.1107/S2052252515008337 (PMC4491315; doi:10.1107/S2052252515008337)
Supplement: Supplementary file 1 [file m-02-00431-sup1.pdf]

IUCrJ

Volume 2 (2015)

Supporting information for article:

Native SAD is maturing

John P. Rose, Bi-Cheng Wang and Manfred S. Weiss

Table S1 A listing of Native-SAD structures by year

Year	Molecule	Uniprot ID	PDB ID	[1]MWT (SEQRES)	[2]NCS	Source	λ (Å)	[3]Anomalous Scatters (ASU)	[4]Bijvoet Ratio	[5]Reference
1981	Crambin	P01542	1CRN	4.736	1	Cu Ka	1.5418	6 S	1.51	DOI: 10.1038/290107a0
1999	Lysozyme	P00698	1LZ8	14.313	1	NSLS X9B	1.5400	10 S, 8 Cl , 1 Na	2.40	DOI: 10.1006/jmbi.1999.2743
2000	Obelin	Q27709	1EL4	22.225	1	APS 17ID	1.7400	8 S, 1 Cl	1.21	DOI: 10.1110/ps.9.11.2085
2001	IGF2R Domain 11	P11717	1GP0	15.547	1	ESRF BM14	1.7700	11 S	1.44	DOI: 10.1093/emboj/21.5.1054
2001	DNA 5-mer		1ICK	1.872	2	NSLS X8C	1.5400	10 P, 1 Mg	1.88	DOI: 10.1107/S0907444901006382
2001	α-crustacyanin C1	P80029	1I4U	20.668	2	ESRF BM14	1.7700	14 S	1.00	DOI: 10.1107/S0907444901009362
2001	Apocrustacyanin A1	P80029	1H91	20.537	2	SRS 9.5	2.0450	12 S	1.19	DOI:10.1107/S0907444901009350
2002	F53F4.3 CAP-Gly	Q20728	1LPL	10.345	1	APS 17ID	1.7400	4 S	0.99	DOI: 10.1074/jbc.M208512200
2002	Tryparedoxin form I	O77093	1O6J	16.909	2	ESRF ID14-2	1.7700	14 S	1.10	DOI: 10.1107/S0907444901016808
2002	Tryparedoxin form II	O77093	1O81	17.143	2	ESRF BM14	1.7700	29 S	1.57	DOI: 10.1107/S0907444901016808
2002	LB trypsin inhibitor	P01056	1H34	9.097	1	Cu Ka	1.5418	14 S	1.66	DOI: 10.1107/S0907444902020917
2002	PA Lectin-1	Q05097	1L7L	12.762	1	APS 17ID	1.5400	3 S, 1 Ca	1.48	DOI: 10.2210/pdb1l7l/pdb
2003	Sso10a	Q92838	1R7J	11.083	1	Cr Ka	2.2900	8 S	2.31	DOI: 10.1016/j.jmb.2004.05.044
2003	PRRSV	Q9YJI1	1P65	8.157	2	DESY BW7A	1.7400	8S	1.11	DOI: 10.1016/j.str.2003.09.018
2003	Viscotoxin A3	P01538	1OKH	4.835	2	Cu Ka	1.5418	13 S, 2 P	2.02	DOI: 10.1107/S0907444903018973
2003	Bubble protein	P83799	1UOY	6.570	1	Cu Ka	1.5418	8 S	1.48	DOI: 10.1107/S0907444903025927
2003	PA1204	Q9I4D4	1RTT	21.094	1	NSLS X12C	1.7000	4 S	0.69	DOI: 10.1107/S0907444906001600
2003	PF1951	Q8TZN6	1NNH	33.991	1	Cr Ka	2.2900	8 S, 1 Na	1.43	DOI: 10.2210/pdb1nnh/pdb
2004	GGACT	Q923B0	1VKB	18.534	1	ALS 8.3.1	1.7400	8 S	1.04	DOI: 10.1002/prot.20610
2004	Coprogen oxidase	P11353	1TK1	29.894	1	Cr Ka	2.2900	8 S	1.41	DOI: 10.1074/jbc.M406050200
2004	svPLA2 homolog	P49121	1S8I	14.023	1	Cu Ka	1.5418	17 S	1.47	DOI: 10.1074/jbc.M410588200
2004	CIB	Q99828	1XO5	41.804	2	Cr Ka	2.2900	8 S, 8 Ca	2.70	DOI: 10.1074/jbc.M411515200
2004	AF2228	O28055	1SAU	13.477	1	ELETTRA	1.9000	5 S	1.19	DOI: 10.1107/S0907444904003002
2004	Phospholipase A2	P00593	1VKQ	13.804	1	NSLS X9A	1.5400	18 S, 1 Ca, 1 Cl	2.75	DOI: 10.1107/S090744490401697X
2004	Aequorin 1	P07164	1SL8	21.861	1	Cr Ka	2.2900	8 S, 3 Ca	3.87	DOI: 10.1110/ps.041142905
2004	VC2159	Q9KQ45	1U8S	21.366	2	NSLS X12C	1.7000	20 S	1.08	DOI: 10.2210/pdb1u8s/pdb
2004	Q15691	Q15691	1VKA	17.370	2	Cr Ka	2.2900	24 S	1.60	DOI: 10.2210/pdb1vka/pdb
2004	GTP BP	Q5SM23	1WF3	33.809	2	Cr Ka	2.2909	12 S, 3 P, 1 Mg	1.71	DOI: 10.2210/pdb1wf3/pdb
2004	YkuL	O31698	1YAV	17.988	2	NSLS X12C	1.7000	15 S	1.02	DOI:10.2210/pdb1yav/pdb
2005	FKBP35	Q8I4V8	2FBN	22.997	2	Cr Ka	2.2900	8 S	1.14	DOI: 10.1002/pro.226

Year	Molecule	Uniprot ID	PDB ID	[1]MWT (SEQRES)	[2]NCS	Source	λ (Å)	[3]Anomalous Scatters (ASU)	[4]Bijvoet Ratio	[5]Reference
2005	Mud-1	Q10256	1Z96	4.059	2	ESRF BM14	1.7710	4 S	1.20	DOI: 10.1038/sj.emboj.7600797
2005	SusB	G8JZS4	2D73	84.309	2	Cr Ka	2.2900	56 S, 2 Ca	2.22	DOI: 10.1074/jbc.M806115200
2005	ToxA	P78737	1ZLD	13.213	1	Cu Ka	1.5418	4 S	0.74	DOI: 10.1105/tpc.105.034918
2005	FGE protein	Q8NBK3	1Z70	34.863	1	Cu Ka	1.5418	12 S, 2 Ca, 1 Cl	1.81	DOI: 10.1107/S0907444905013831
2005	AF1432	O28840	1YNB	18.066	1	Cr Ka	2.2900	6 S	1.57	DOI: 10.2210/pdb1ynb/pdb
2005	PA1835	Q9I2R0	1YOC	16.012	2	Cr Ka	2.2900	18 S	2.04	DOI: 10.2210/pdb1yoc/pdb
2005	AF0625	O29630	1YQE	31.927	1	Cr Ka	2.2900	7 S, 2 P	1.81	DOI: 10.2210/pdb1yqe/pdb
2005	PF0523	Q8U3E5	1ZD0	16.823	1	Cr Ka	2.2900	3 S, 1 Mg	1.37	DOI: 10.2210/pdb1zd0/pdb
2005	PA1268	Q9I476	2AZP	33.866	1	Cr Ka	2.2900	10 S	1.48	DOI: 10.2210/pdb2azp/pdb
2005	PHS023	O73983	2CVI	9.667	2	Cr Ka	2.2900	28 S, 2 K	4.89	DOI: 10.2210/pdb2cvi/pdb
2005	TTHA1634	Q5SHU6	2D5W	68.624	2	Cr Ka	2.2900	44 S	1.54	DOI: 10.2210/pdb2d5w/pdb
2006	Proteinase K	P06873	2ID8	28.934	1	APS 22 ID	0.9800	10 S, 1 Ca, 1 Cl	0.67	10.1107/S0907444906038534
2006	DIPP-1	O95989	2FVV	22.023	1	Cu Ka	1.5418	5 S, 6 P, 1 Cl	1.47	DOI: 10.1002/prot.22489
2006	DUSP9	Q99956	2HXP	17.656	1	NSLS X12C	1.7000	3 S, 1 P	0.95	DOI: 10.1007/s10969-007-9036-1
2006	Majastridin	P05449	2NXV	28.808	2	MAXLAB I911	1.8000	24 S	1.13	DOI: 10.1016/j.bbapap.2007.11.005
2006	SCO7518	Q93J02	2DG8	21.941	4	Cr Ka	2.2900	8 S	0.82	DOI: 10.1016/j.febslet.2014.09.037
2006	Fibronectin	P02751	2CG6	10.106	1	ESRF BM14	1.7700	11 S	1.79	DOI: 10.1016/j.jmb.2007.02.061
2006	CBP1	C9V488	2HQ8	20.829	2	APS 22ID	1.7000	14 S, 6 Ca	2.29	DOI: 10.1039/b716535h
2006	A2R (VEGF-E)	P52584	2GNN	14.249	4	SLS X06SA	1.7000	46 S, 3 Cl	1.88	DOI: 10.1074/jbc.M601842200
2006	Lam16A	Q874E3	2CL2	31.900	1	ESRF ID14-4	1.7800	13 S	1.09	DOI: 10.1107/S0907444906036407
2006	NBR1 PB1	Q14596	2G4S	9.807	1	DESY X12	2.0000	4 S, 1 Cl	2.21	DOI: 10.1107/S0907444906055624
2006	hARH3	Q9NX46	2G4K	37.731	1	DESY X12	2.0000	15 S, 2 Cl, 2 Mg	2.12	DOI: 10.1107/S0907444906055624
2006	HNL	P52704	2G4L	29.227	1	DESY X12	2.0000	13 S, 1 Cl	1.92	DOI: 10.1107/S0907444906055624
2006	Insulin	P01315	2G4M	5.765	1	DESY X12	2.0000	6 S	2.18	DOI: 10.1107/S0907444906055624
2006	LeuB	P9WKK9	2G4O	35.393	4	DESY X12	2.0000	26 S, 6 Cl	1.46	DOI: 10.1107/S0907444906055624
2006	MogA	O53877	2G4R	16.188	3	DESY X12	2.0000	6 S, 1 Cl	1.13	DOI: 10.1107/S0907444906055624
2006	PPE	P00772	2G4T	25.908	1	DESY X12	2.0000	12 S, 1 Na	1.55	DOI: 10.1107/S0907444906055624
2006	RNAse A	P61823	2G4W	13.690	2	DESY X12	2.0000	25 S, 1 Cl	2.55	DOI: 10.1107/S0907444906055624
2006	Thaumatin	Q8RVT0	2G4Y	22.219	1	DESY X12	2.0000	17 S	1.87	DOI: 10.1107/S0907444906055624
2006	Trypsin P1	P35049	2G51	22.186	1	DESY X12	2.0000	7 S, 3 Cl	2.18	DOI: 10.1107/S0907444906055624
2006	Titin	Q8WZ42	2ILL	21.607	1	DESY X12	2.0000	4 S, 2Cl	1.73	DOI: 10.1107/S0907444906055624
2006	ATU299	A9CI22	2HLY	22.748	1	Cr Ka	2.2900	10 S	1.81	DOI: 10.2210/pdb2hly/pdb
2006	RSP_3884	Q3HKK5	2HZG	42.457	2	NSLS X12C	1.7400	52 S	1.24	DOI: 10.2210/pdb2hzg/pdb
2006	PTO0218	Q6L2J9	2I52	13.760	6	NSLS X4A	1.7430	30 S, 12 Ca, 1 Cl	2.68	DOI: 10.2210/pdb2i52/pdb
2007	TP0655	O83661	2V84	39.090	1	Cu Ka	1.5418	12 S, 5 Cl	1.28	DOI: 10.1016/j.jmb.2007.08.018

Year	Molecule	Uniprot ID	PDB ID	[1]MWT (SEQRES)	[2]NCS	Source	λ (Å)	[3]Anomalous Scatters (ASU)	[4]Bijvoet Ratio	[5]Reference
2007	GITRL	Q7TS55	2QDN	14.993	2	NSLS X4A	1.7432	12 S	1.06	DOI: 10.1073/pnas.0710529105
2007	ASP1	Q8WRW5	3BJH	13.186	1	ESRF BM14	1.7700	11 S	1.56	DOI: 10.1074/jbc.M311212200
2007	CD5 domain III	P06127	2JA4	11.301	1	SLS X06SA	1.8000	8 S	1.48	DOI: 10.1074/jbc.M611699200
2007	R-PTP-N	Q16849	2QT7	9.829	2	NSLS X6A	1.5895	2 S, 3 Ca	1.71	DOI: 10.1074/jbc.M708144200
2007	phoP	P23836	2E6U	16.721	1	Cr Ka	2.2900	8 S, 3 P, 1 Ca, 3 Cl	5.70	DOI: 10.1107/S0907444905012734
2007	SCO0332	Q9RK47	2ZB9	23.596	2	Cr Ka	2.2909	4 S	0.79	DOI: 10.1107/S0907444907059835
2007	SCO5068	Q9ADD9	2REK	41.696	2	Cr Ka	2.2900	6 S	0.52	DOI: 10.2210/pdb2rek/pdb
2007	PH1780	O59416	2YZQ	31.906	1	Cr Ka	2.2900	9 S	1.45	DOI: 10.2210/pdb2yzq/pdb
2007	TTHA1012	Q5SJJ6	2YZY	22.592	1	Cr Ka	2.2900	2 S	0.81	DOI: 10.2210/pdb2yzy/pdb
2007	SCO7815	Q9FBX0	2ZCX	25.366	1	Cr Ka	2.2909	4 S	1.08	DOI: 10.2210/pdb2zcx/pdb
2008	AF1514	O28758	3C0F	10.505	1	Cr Ka	2.2900	3 S	1.46	DOI: 10.1002/prot.22025
2008	Npun_R1517	B2IZS7	3E56	12.895	1	Cu Ka	1.5418	3 S	0.65	DOI: 10.1002/prot.22308
2008	Antifreeze protein	A0ZT93	2ZIB	14.500	1	S8 BL44B2	1.7000	14 S	1.56	DOI: 10.1016/j.jmb.2008.07.042
2008	Plbd2	Q3TCN2	3FBX	63.123	1	BESSY 14.2	1.9000	22 S, 1 Na, 0.05 XE	1.90	DOI: 10.1016/j.jmb.2009.03.077
2008	All3740	Q8YQS9	3DU1	27.740	1	Cu Ka	1.5418	6 S	0.62	DOI: 10.1016/j.jsb.2008.09.010
2008	Takeout protein	B5ABT1	3E8T	24.479	1	Cu Ka	1.5418	8 S	0.76	DOI: 10.1074/jbc.M807467200
2008	Viscotoxin A1	D0VWT3	3C8P	4.889	2	Cu Ka	1.5418	12 S	1.48	DOI: 10.1107/S0907444908022646
2008	TTHA0621	Q5SKM2	2ZGI	27.252	4	Cr Ka	2.2909	17 S	1.08	DOI: 10.1107/S1744309109050052
2008	FeoA	D0VWU5	3E19	7.972	4	APS 22ID	1.9000	20 S, 1 P	1.82	DOI: 10.2210/pdb3e19/pdb
2008	ATV ORF131	Q3V4Q3	3FAJ	16.390	1	SOLEIL Proxma 1	2.0000	3 S, 3 Cl	2.05	DOI: 10.2210/pdb3faj/pdb
2009	Lipase	Q7LST4	3G7N	27.289	2	APS 22ID	1.9000	23 S	1.27	DOI: 10.1002/prot.22676
2009	PRKAR1A	P00514	3IM3	5.995	1	APS 23ID	1.9000	3 S	1.39	DOI: 10.1016/j.str.2009.12.012
2009	GFP	P42212	2WUR	26.854	1	Cu Ka	1.5418	6 S	0.63	DOI: 10.1021/ja1010652
2009	PLA2-II	Q9XG81	2WG7	13.965	2	ESRF BM14	1.7700	28 S, 2 Ca, 1 Na	2.83	DOI: 10.1074/jbc.M109.008466
2009	IYD protein	Q9DCX8	3GB5	29.940	1	AOS 24-ID-C	1.6530	7 S, 2 P	1.03	DOI: 10.1074/jbc.M109.013458
2009	DegV	P32436	3FYS	35.124	1	Cr Ka	2.2900	17 S	1.90	DOI: 10.1107/S0907444909007756
2009	Protein P	Q0GBY3	3L32	5.339	2	ESRF BM14	1.7700	6 S	1.28	DOI: 10.1128/JVI.02557-09
2009	TPHE39A		2ZY6	10.273	1	PF AR-NW12A	1.7000	38 P, 7 Ca, 1 Mg, 1 Cl	6.14	DOI: 10.1261/rna.1614709
2009	NGAL	P80188	3HFW	39.506	1	ESRF ID29	1.8500	15 S, 2 P, 1 Mg, 1 K , 1 Cl	2.49	DOI: 10.2210/pdb3hfw/pdb
2009	BH3703	Q9K6M5	3I0T	21.873	2	APS 31ID	2.2700	11 S	1.32	DOI: 10.2210/pdb3i0t/pdb
2010	HP0721	O25423	2XRH	11.490	1	ESRF ID29	1.9100	7 S	1.53	DOI: 10.1002/prot.22988
2010	GluRS	O26157	3AII	63.089	1	Cr Ka	2.2900	16 S, 1 Ca, 1 Zn	2.54	DOI: 10.1093/nar/gkq605
2010	AF1382	O28889	3O3K	11.144	1	APS 22ID	1.9000	4 S	1.17	DOI: 10.1107/S0907444912026212
2010	PA1645	Q9I380	2XU8	13.102	3	DIAMOND I03	1.6000	17 S	0.94	DOI: 10.1107/S1744309112044739
2010	TDRD3	Q9H7E2	3PMT	6.876	1	Cu Ka	1.5418	2 S	0.72	DOI: 10.1371/journal.pone.0030375

Year	Molecule	Uniprot ID	PDB ID	[1]MWT (SEQRES)	[2]NCS	Source	λ (Å)	[3]Anomalous Scatters (ASU)	[4]Bijvoet Ratio	[5]Reference
2010	TorS/TorT	Q87ID1	3O1I	31.666	4	NSLS X4A	1.7400	28 S	0.75	doi:10.1107/S0907444913001479
2011	TNFRSF21	O75509	3U3Q	34.157	1	PF BL17A	2.0000	21 S	1.67	DOI 10.1107/S0907444912004490
2011	hPAR17	Q9Y237	3UI5	11.143	1	Cu Ka	1.5418	15 S	1.55	DOI: 10.1021/ja2086195
2011	Lectin SML2	P81860	2YIL	15.077	6	DESY X12	2.0000	105 S, 7 Cl	3.04	DOI: 10.1107/S0907444911037796
2011	TNFRSF21	O75509	3U3V	34.157	1	PF BL1A	2.7000	21 S	2.82	DOI: 10.1107/S0907444912004490
2011	dntR protein	Q7WT50	2Y7P	24.813	1	ESRF ID23	1.8200	12 S	1.25	DOI: 10.1111/j.1365-2958.2011.07673.x
2011	RYR-1	P11716	3RQR	26.433	1	Cr Ka	2.2909	4 S	1.06	DOI: 10.1111/j.1742-4658.2012.08755.x
2011	A654L	O41136	3QB8	22.397	2	NSLS X6A	1.7000	21 S, 3 P	1.41	DOI: 10.2210/pdb3q88/pdb
2011	Netrin-G2	Q96CW9	3TBD	38.923	1	NSLS X4A	1.7400	26 S, 1P, 1 Ca	1.87	doi:10.1107/S0907444913001479
2011	cysZ	Q5QUJ8	3TX3	28.942	2	NSLS X4A	1.7400	41 S, 4 Cl	1.66	doi:10.1107/S0907444913001479
2011	RPA2292	Q6N7G5	3VA9	18.727	1	NSLS X4A	1.7400	3 S, 1 Cl	0.92	doi:10.1107/S0907444913001479
2012	cxl34b.11	F1Q6N2	4HCS	9.299	1	Cr Ka	2.2900	7 S	2.36	DOI: 10.1002/prot.24380
2012	Saposin-A	P07602	4DDJ	9.054	1	Cr Ka	2.2900	9 S	2.72	DOI: 10.1073/pnas.1115743109
2012	cofA	Q59393	3VOR	18.886	1	SP8 BL38B1	1.5000	5 S	0.67	DOI: 10.1107/S0907444912034464
2012	Glutaredoxin	Q9NLB2	4HJM	12.418	1	Cu Ka	1.5418	6 S	0.93	DOI: 10.1107/S1399004713025285
2012	S100-A2	P29034	4DUQ	11.068	2	SLS X06SA	0.9000	6 S, 4 Ca	0.73	DOI: 10.1111/j.1742-4658.2012.08556.x
2013	UmAbf62A	Q4P6F4	4N1I	37.014	1	Cu Ka	1.5418	8 S, 1 Ca	1.13	DOI 10.1074/jbc.M113.528133
2013	FAM3B	Q9D309	2YOP	22.127	3	ESRF ID29	2.0000	24 S	1.28	DOI: 10.1016/j.str.2012.12.009
2013	NTPDase 1	Q5ZUA2	4BRM	41.844	2	BESSY BL14.2	1.9000	32 S, 2 Cl	1.59	DOI: 10.1016/j.str.2013.05.016
2013	MGST1-L1	O14684	4BPM	20.328	1	SLS X06DA	2.0660	9 S, 2 Cl	2.34	DOI: 10.1021/cg500157x
2013	CNOT1	P26651	4J8S	25.739	1	Cu Ka	1.5418	9 S	0.79	DOI: 10.1038/nsmb.2572
2013	Ld lectin	A7UNK4	4NDS	10.282	2	ESRF BM14	1.6980	12 S, 3 Na	1.35	DOI: 10.1093/glycob/cwu136
2013	Carwin	U5HK42	4JP6	13.665	1	Cu Ka	1.5418	6 S	0.89	DOI: 10.1107/S0907444913018015
2013	PilO2Bp	Q63JW5	4BYZ	22.167	1	ESRF BL14	1.7710	4 S, 1 P, 5 K	2.55	DOI: 10.1371/journal.pone.0094981
2013	Milk protein	Q6SVB6	4NYR	18.725	1	PF BL1A	2.7000	5 S	1.86	DOI: 10.2210/pdb4nyr/pdb
2013	DnaK	P0A6Y8	4JN4	65.813	2	NSLS X4B/C	1.7400	43 S, 6 P, 2 Mg	1.23	doi:10.1107/S0907444913001479
2014	CopN	Q9Z8L4	4P3Z	46.532	1	SOLEIL Proxma 1	1.9040	12 S	0.99	DOI: 10.1074/jbc.M114.568436
2014	TM0633	Q9WZA1	4QDN	15.826	1	ESRF ID23-EH1	2.0600	8 S, 1	2.05	DOI: 10.1093/glycob/cwu113
2014	Z DNA duplex		4OCB	3.727	1	APS 22ID	1.5400	11 P	1.75	DOI: 10.1107/S1399004714004684
2014	YetJ	O31539	4TKQ	24.100	1	NSLS X4A	2.0700	8 S, 1 Ca, 3 Cl	3.27	DOI: 10.1107/S1399004714013376
2014	ThiT	S5L6I0	4TKR	24.052	2	NSLS X4A	2.0737	18 S, 4 P	1.90	DOI: 10.1107/S1399004714013376
2014	EGFRK	P00533	4TKS	37.660	1	NSLS X4A	2.0710	17 S	2.42	DOI: 10.1107/S1399004714013376
2014	HCV nE1	H9XGD6	4UOI	9.606	6	DIAMOND I04	1.7712	24 S	1.10	DOI: 10.1107/S139900471401339X
2014	NS1	Q5SBG8	4TPL	42.556	2	APS 23ID	1.7462	35 S	1.07	DOI: 10.1107/S1399004714017556
2014	AggA	G5TBZ9	4PH8	16.746	2	ESRF BM14	1.7500	6 S	0.71	DOI: 10.1371/journal.ppat.1004404

Year	Molecule	Uniprot ID	PDB ID	[1]MWT (SEQRES)	[2]NCS	Source	λ (Å)	[3]Anomalous Scatters (ASU)	[4]Bijvoet Ratio	[5]Reference
2014	Muskelin	Q99PV3	4OYU	23.146	2	BESSY 14.1	1.9000	18 S	1.22	DOI:10.1016/j.str.2014.11.016
2014	PF0904	Q8U2D2	4PGO	12.677	1	SLS X06SA	2.0660	3 S, 1 Cl	1.85	DOI:10.1038/nmeth.3211
2014	PF0907	Q8U2D5	4PII	29.252	1	SLS X06SA	2.0660	8 S, 12 Cl	2.89	DOI:10.1038/nmeth.3211
2014	JEV protease	O90417	4R8T	17.617	1	SLS X06SA	2.0660	5 S	1.20	DOI:10.1038/nmeth.3211
2014	Pol IV - DNA	Q47155	4R8U	48.417	2	SLS X06SA	2.0660	28 S, 75 P, 2 Ca	3.50	DOI:10.1038/nmeth.3211
2014	TTC1385	Q72HU9	4TN8	12.461	1	SLS X06SA	2.0660	5 S, 6 Cl	3.28	DOI:10.1038/nmeth.3211
2014	ACD11	O64587	4TNO	9.978	1	SLS X06SA	2.0660	3 S, 2 Cl	2.43	DOI:10.1038/nmeth.3211
2014	mPGES1	B5MCC3	4WAB	20.328	1	SLS X06SA	2.0660	9 S, 2 Cl	2.53	DOI:10.1038/nmeth.3211
2014	CENP-M	Q9NSP4	4WAU	19.220	2	SLS X06SA	2.0660	14 S	1.36	DOI:10.1038/nmeth.3211
2014	PF1771	Q8U046	4WBX	44.038	1	SLS X06SA	2.0660	13 S	1.23	DOI:10.1038/nmeth.3211
2014	TAF8/TAF10 Complex	Q12962/ Q7Z7C8	4WV4	22.000	1	SOLEIL Proxma 1	1.9075	8 S, 1 Cl	1.70	DOI:10.1038/ncomms7011
2014	Tubulin-Stathmin-TTL Complex	P81947/ Q6B856/ P63043/ E1BQ43	4WBN	261.249	1	SLS X06SA	2.0660	118 S, 13 P, 3 Ca, 2 Cl	2.69	DOI:10.1038/nmeth.3221
2015	Philin	Q8EII5	4US7	9.843	2	BESSY 14.1	1.77	2 S, 1 Na	0.99	DOI: 10.1107/S1399004715003272

[1]Molecular weight for the molecule as reflected by the PDB SEQRES records for the PDB entry. The EXExPASy - ProtParam tool was use to calculate the molecular weight based on the PDB fasta sequence for the chain.

[2]The number of molecules in the crystallographic asymmetric unit as reflected in the PDB entry.

[3]The number and type of anomalous scatterer found in the PDB entry as reflected in the ATOMS and FORMUL records.

[4]The Bijvoet ratio [(<|F|>)(<F>)$^{-1}$] for the PDB entry (Hendrickson, W. A. & Teeter, M. M. (1981). *Nature* **290**, 107-113). The values were calculated using James Holton's xtalsize server http://bl831.als.lbl.gov/xtalsize.html.

[5]The Digital Object Identifier (DOI) of the paper or PDB entry describing the work.
